# Increasing HIV testing and linkage to care among men in rural South Africa using conditional financial incentives and a decision support app: A process evaluation

**DOI:** 10.1371/journal.pgph.0003364

**Published:** 2024-06-18

**Authors:** Thulile Mathenjwa, Luchuo Engelbert Bain, Oluwafemi Adeagbo, Hae-Young Kim, Maxime Inghels, Thembelihle Zuma, Sally Wyke, Maryam Shahmanesh, Nuala McGrath, Ann Blandford, Philippa Matthews, Dickman Gareta, Manisha Yapa, Till Bärnighausen, Frank Tanser, Janet Seeley

**Affiliations:** 1 Africa Health Research Institute, Durban, South Africa; 2 Department of Psychology, Faculty of Humanities, University of Johannesburg, Johannesburg, South Africa; 3 International Development Research Centre, IDRC, Ottawa, Canada; 4 Department of Community and Behavioral Health, College of Public Health, University of Iowa, Iowa City, IA, United States of America; 5 Department of Sociology, Faculty of Humanities, University of Johannesburg, Johannesburg, South Africa; 6 Department of Population Health, New York University Grossman School of Medicine, New York, NY, United States of America; 7 Lincoln International Institute for Rural Health, University of Lincoln, Lincoln, United Kingdom; 8 Centre Population et Développement (UMR 196 Paris Descartes–IRD), SageSud (ERL INSERM 1244), Institut de Recherche pour le Développement, Paris, France; 9 University of KwaZulu-Natal, Durban, South Africa; 10 Division of Infection and Immunity, University College London, London, United Kingdom; 11 School of Social and Political Sciences, University of Glasgow, Glasgow, United Kingdom; 12 Institute for Global Health, University College London, London, United Kingdom; 13 Department of Social Statistics and Demography, Faculty of Social Sciences, University of Southampton, Southampton, United Kingdom; 14 University College London Interaction Centre, University College London, London, United Kingdom; 15 The Kirby Institute, University of New South Wales, Sydney, Australia; 16 Heidelberg Institute of Global Health (HIGH), Heidelberg University, Heidelberg, Germany; 17 Centre for Epidemic Response and Innovation, School for Data Science and Computational Thinking, Stellenbosch University, Cape Town, South Africa; 18 Department of Global Health and Development, London School of Hygiene and Tropical Medicine, London, United Kingdom; South African Medical Research Council, SOUTH AFRICA

## Abstract

Men in sub-Saharan Africa are less likely to accept HIV testing and link to HIV care than women. We conducted a trial to investigate the impact of conditional financial incentives and a decision support application, called EPIC-HIV, on HIV testing and linkage to care. We report the findings of the trial process evaluation to explore whether the interventions were delivered as intended, identify mechanisms of impact and any contextual factors that may have impacted the trial outcomes. Between August 2018 and March 2019, we conducted in-depth interviews and focus group discussions with trial participants (n = 31) and staff (n = 14) to examine views on the implementation process, participant responses to the interventions and the external factors that may have impacted the implementation and outcomes of the study. Interviews were audio-recorded, transcribed, and translated where necessary, and thematically analyzed using ATLAS-ti and NVivo. Both interventions were perceived to be acceptable and useful by participants and implementers. EPIC-HIV proved challenging to implement as intended because it was difficult to ensure consistent use of earphones, and maintenance of privacy. Some participants struggled to navigate the EPIC-HIV app independently and select stories that appealed to them without support. Some participants stopped exploring the app before the end, resulting in an incomplete use of EPIC-HIV. While the financial incentive was implemented as intended, there were challenges with eligibility. The convenience and privacy of home testing influenced the uptake of HIV testing. Contextual barriers including fear of HIV stigma and disclosure if diagnosed with HIV, and expectations of poor treatment in clinics may have inhibited linkage to care. Financial incentives were relatively straightforward to implement and increased uptake of home-based rapid HIV testing but were not sufficient as a ‘stand-alone’ intervention. Barriers like fear of stigma should be addressed to facilitate linkage to care.

## Introduction

Men in sub-Saharan Africa are less likely than women to test for HIV, initiate HIV treatment, or remain in care [1–4]. Some of the reasons for this poor uptake of HIV services are lack of motivation or information [5] and prioritization of current circumstances (e.g., opportunity to earn a livelihood) over future issues (e.g., going to the clinic to refill HIV treatment—health) [6]. For example, in the treatment as prevention (TasP) trial (ANRS 12249) conducted in KwaZulu-Natal, South Africa, some of the men who did not consent to HIV testing, reported feeling healthy as a reason for being reluctant to test [1].

We investigated the effect of financial micro-incentives and an HIV specific decision support app (called EPIC-HIV [Empowering People through Informed Choices for HIV]) in the Home-based Intervention to Test and Start (HITS) trial (ClinicalTrials.gov # NCT03757104). Both interventions were intended to increase uptake of HIV testing and HIV treatment among men to reduce population viral load and ultimately lower HIV incidence among young women [7]. The financial micro-incentives sought to address present bias by providing immediate external reward (R50 food voucher) contingent on first, participating in the HIV rapid test and second, if found to be living with HIV, presenting to a local clinic for HIV care. The content of the EPIC-HIV app [8] drew on self-determination theory [9], seeking to shift men’s motivation for HIV testing and linkage to care from unmotivated to internally motivated by changing perceptions of the benefits of ART [8]. It did this by offering men the opportunity to explore content rooted in local narratives, from the perspective of young, middle aged and older men. We intended to provide a form of experiential information to increase risk perception, salience and likelihood of response [8]. The experiential information included examples of how other men living with HIV navigated the barriers to care and what they found to be the benefits of testing early, linking to care and initiating antiretroviral therapy. Two apps were developed, EPIC-HIV 1 aimed at increasing rates of home-based HIV testing and EPIC-HIV 2 was to increase linkage to care amongst those found to be living with HIV. The apps used a mixture of audio, text, video, still photos and graphics. The implementation of both interventions is described below.

An outcome evaluation of the HITS trial showed that the financial incentives, which were offered to both men and women, successfully increased HIV testing for both but only increased linkage to care among women [10, 11]. EPIC-HIV did not have an effect on either testing or linkage to care [10, 11]. We conducted a process evaluation to understand whether the interventions were delivered as intended, identify mechanisms of impact and contextual factors that may have impacted the trial outcomes using in-depth interviews and focus group discussions with trial staff and participants. A process evaluation is particularly useful in explaining why an intervention had no effect in a trial, helping to discern conceptual failure, poor implementation, and contextual barriers.

## Methods

### Setting

The study was conducted in uMkhanyakude district in northern KwaZulu-Natal. In this area the Africa Health Research Institute (AHRI) has maintained a large population-based demographic and HIV surveillance study since 2003. It has included the offer of annual home-based testing for HIV since 2017. The study area is predominately rural and poor [12] with high unemployment rates (62% of adults without formal employment) [13]. HIV prevalence was estimated as 19% among men and 40% among women who provided an anonymized sample (collected using dry blood spots) in 2018 [13].

### Implementation of the HITS interventions

**Fig 1:**
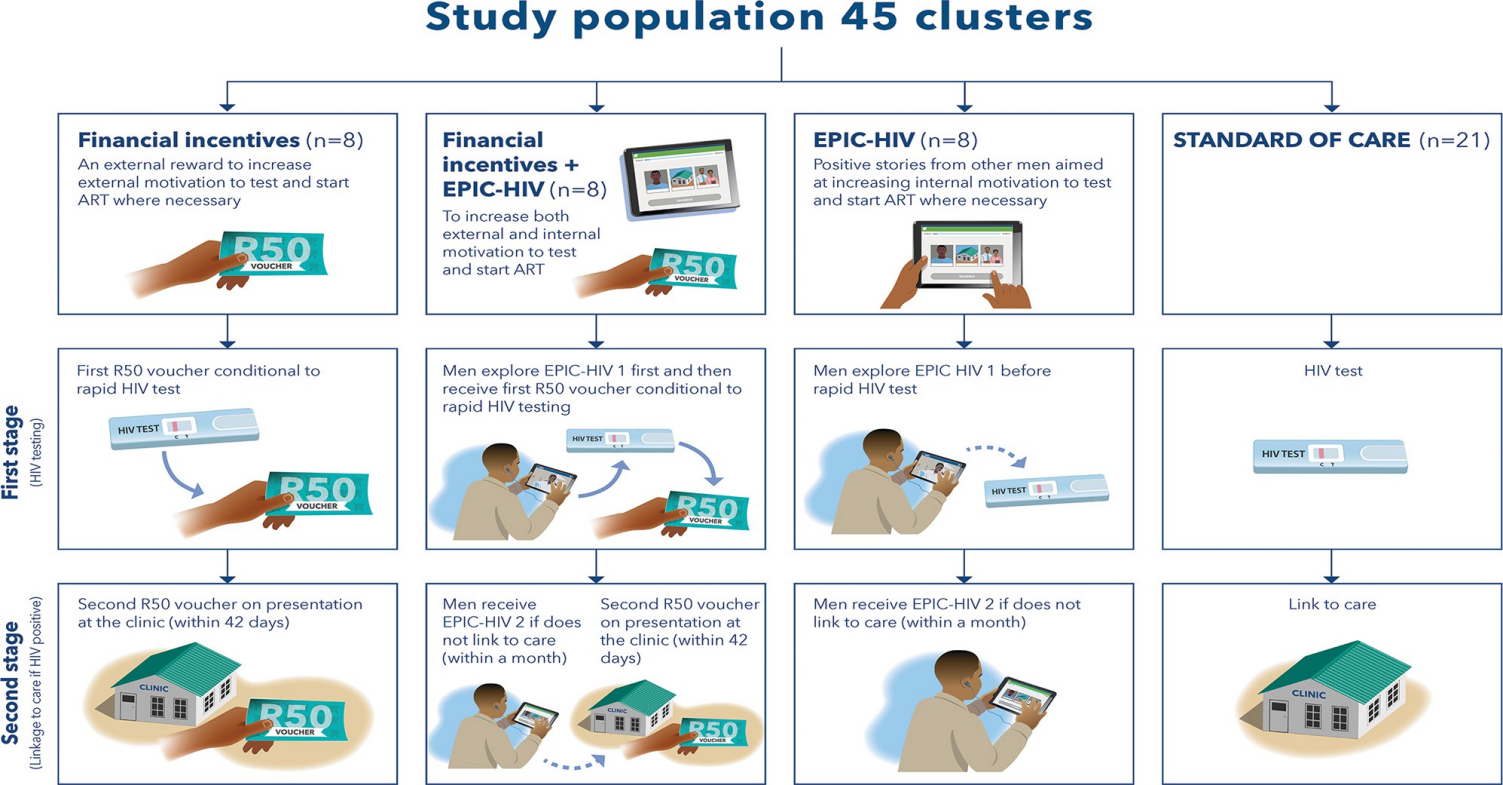
HITS interventions delivered to 45 communities in four clusters.

The HITS trial was a cluster randomized trial conducted in 45 communities (clusters) in uMkhanyakude between 2018–2021, built on the AHRI population-based HIV testing platform [7]. Fig 1 illustrates that in a 2x2 factorial design, 45 communities (clusters) were assigned to four arms including financial micro-incentives alone, the EPIC-HIV app alone, both financial micro-incentives and the EPIC-HIV app and standard of care.

Participants in the arms including financial micro-incentives were offered a R50 (US$3) food voucher from a local supermarket by AHRI fieldworkers conditional on accepting a home-based rapid HIV test (first stage) during annual visits to offer home based HIV testing. AHRI nurses stationed at the 11 primary health clinics within the AHRI HIV surveillance area offered a second R50 food voucher if they tested positive for HIV and sought treatment within 6 weeks (42 days) of the positive HIV test date (second stage).

Only men were included in the arms receiving the EPIC-HIV app. They were offered EPIC-HIV 1 prior to the offer of an HIV test (first stage). The EPIC-HIV 1 app was installed on the AHRI fieldworker’s tablet which they use for routine survey data collection and offered to participants during the annual visits to offer home based HIV testing. The fieldworker was expected to hand over the tablet with the app opened (start screen) to the user with earphones and give them space to allow the user to independently explore the app in private. EPIC-HIV 1 offered different pathways which the participant could navigate, based on whether they were ready to test or not ready to test. The app was designed to be navigated in 5–10 minutes depending on the pathway that the user selected. If participants took a test and tested positive for HIV and did not link to care within 30 days, an experienced AHRI fieldworker visited again, to offer EPIC-HIV 2 installed on their tablet. EPIC-HIV 2 again offered different pathways the user could navigate, with different ‘personalities’ describing the perceived benefits of linkage to care and starting HIV treatment early. EPIC-HIV 2 was designed to be navigated in 10–20 minutes. Both apps were meant to be used once and neither app had an option to quit before the end.

### Process evaluation design

#### Data collection

Our process evaluation was based on qualitative data consisting of in-depth interviews (IDIs) with a purposive sample of men who participated in the HITS trial and AHRI staff who implemented the trial (including fieldworkers, nurses, and a research assistant), and a focus group discussion (FGD) with four AHRI fieldworkers (Table 1). In analyzing these data for our process evaluation, we drew on the UK Medical Research Council (MRC) framework [14] for developing and evaluating complex interventions. The framework suggests three interrelated factors are important in explaining outcomes: 1) implementation–what is implemented and how (whether the intervention was implemented as intended); 2) mechanisms of impact–participants response to and interaction with the intervention; and 3) context–how contextual features shape implementation or how the intervention may work. We describe how we assess these factors using our qualitative data when we describe the data analysis approach below.

**Table 1:** Participants demographics.

Participants	Ages (years)	Sex		Total
		*Female*	*Male*	
HITS trial participants				
Micro-financial incentives only	18–72	0	11	11
Micro-financial incentives and EPIC-HIV	16–65	0	10	10
EPIC-HIV only	20–45	0	10	10
HITS implementers				
Fieldworkers	30–52	4	1	5
Nurses		4	0	4
Tracker (experienced fieldworker who delivered EPIC-HIV 2)		0	1	1
Fieldworkers (FGD)	29–43	0	4	4

The data collection was conducted mid trial from August 2018 to post trial March 2019. Inclusion criteria for the men who participated in the HITS trial were: 1) to have received the HITS interventions (vouchers and/or EPIC-HIV); 2) to have consented to be followed up during the home-based testing visit; 3) to be able to give written informed consent. Inclusion criteria for AHRI staff who implemented the interventions (implementers) were: 1) to have been involved in the implementation of the HITS interventions (vouchers and/or EPIC-HIV); 2) to be willing to be interviewed.

Interviews and the focus group discussion covered similar ground for each group, in an attempt to get different perspectives on the interventions. After general questions about background, they covered views of HIV testing, general views of the two interventions (which were new to the AHRI annual testing regime), views on whether the interventions were successful or not, and why, including what could have been done better and finally views on whether the interventions were likely to be sustainable. Participants were also asked about whether they thought their own behavior or views had been changed.

Participants were recruited by telephone from a predefined list of a random sample of men who participated in the HITS trial and the interviews were conducted in their homes in the main local language (isiZulu). Implementers were recruited face to face and interviewed at AHRI offices using a mixture of English and isiZulu. The interviews and FGD were conducted by a research assistant trained in qualitative methods, familiar with the interventions and setting and lasted between 30 minutes on average. All interviews including FGDs were audio-recorded with permission. At the end of the interview, participants and implementers were offered refreshments.

#### Data analysis

The recorded data were transcribed and translated to English where necessary and the analysis was conducted in English. We used a thematic approach to data analysis, with an initial a priori search for data relevant to the MRC process evaluation framework. Then, following initial familiarization with the data through reading and re-reading transcripts, we looked for codes relevant to implementation, mechanisms of action and context. The data from men who participated in the HITS trial were coded by LEB using ATLAS.ti, and the data from the implementers were separately coded by TM using Nvivo (version 11).

To consider implementation, the extent to which the interventions were delivered as intended, we drew mainly on the views of implementers although some participants also offered views on these aspects of the evaluation. Because our data are qualitative, we could not assess actual fidelity or dose, but we were able to code implementer and participant views and experiences of the ease or difficulty of implementation and whether the full intervention was able to be delivered. To consider mechanisms of action, participants’ response to and interaction with the interventions, we drew mainly on the views and experiences of participants, although on occasion implementers views were also useful. To consider how contextual features shaped implementation of interventions and the actual outcomes, we drew on the views of both participants and implementers to identify external factors that may have facilitated or acted as barriers to the interventions.

### Ethical approval

The study was approved by the Biomedical Research Ethics of the University of KwaZulu-Natal, South Africa (ref number: BFC398/16). The data were collected with the informed consent of participants prior to their participation in the study.

## Results

A total of 45 participants were interviewed: 31 IDIs with men who received the HITS interventions and 10 IDIs and 1 FGD with trial implementers (see Table 1). The HITS trial participants were aged between 16 to 72 years. All the nurses were female.

**Fig 2:**
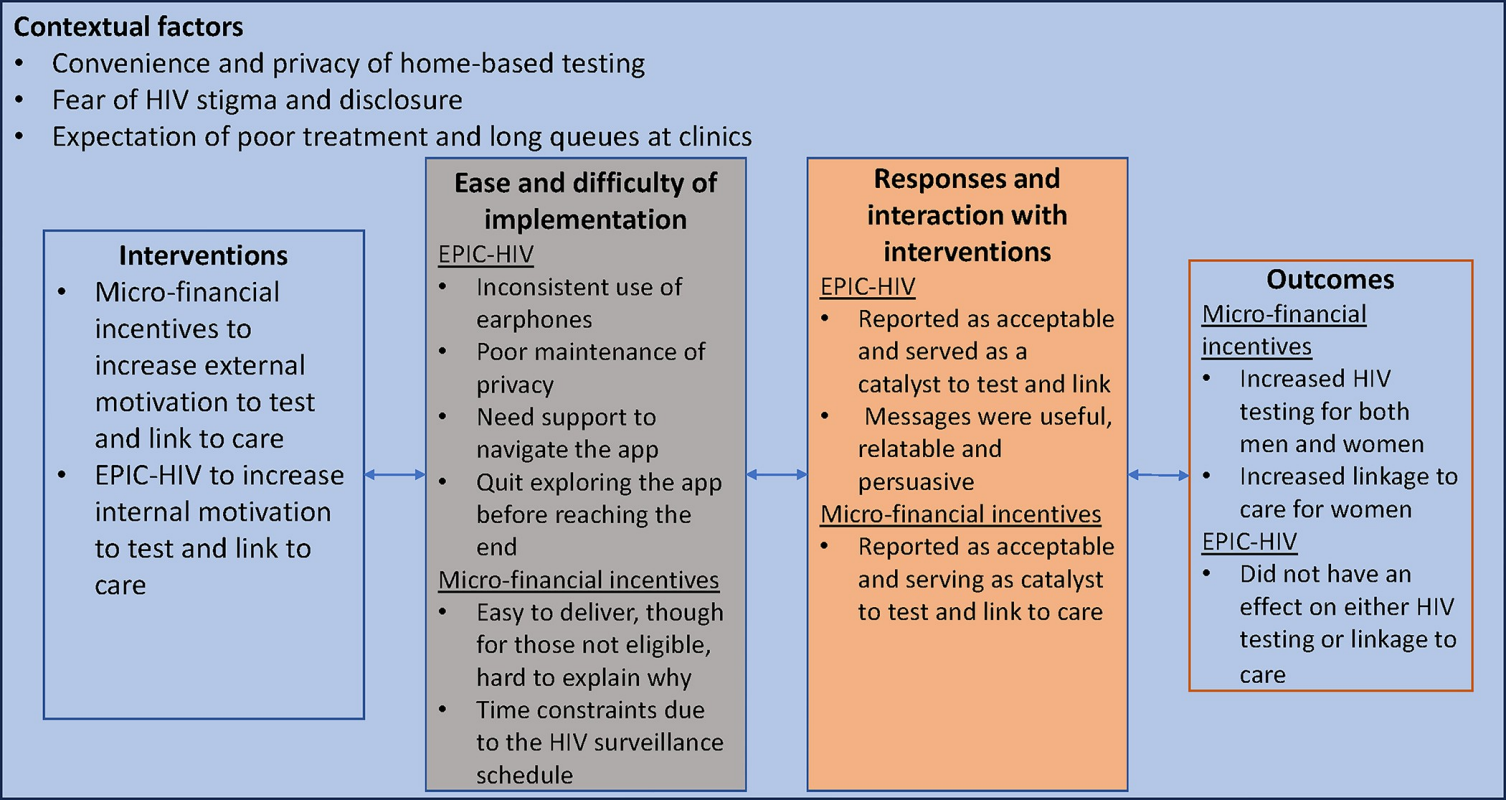
Summary of the findings based on the domains of the key constructs of the MRC framework.

The themes are organized according to the domains of the three interrelated factors of the MRC process evaluation, assessing implementational factors, mechanisms of impact and contextual factors (see Fig 2). Under each theme, we discuss the first stage of implementation followed by the second stage.

### Implementation process

For the 1^st^ stage–HIV testing—the delivery of EPIC-HIV 1 proved to be challenging to implement as intended. For EPIC-HIV 1, participants did not consistently use the earphones. Fieldworkers reported that participants complained about the quality of the earphones which led to some resorting to using the tablet loudspeaker instead. Older men in particular, seemed to struggle to use the earphones and preferred the loudspeaker: *Then*, *the person would say ‘please open loudspeaker for me on the tab’ while we don’t like to do so because it’s important to maintain confidentiality*, *so he can be proud of what he will hear without being heard by another person–*(FGD). Using the loudspeaker, rather than the earphones, could have compromised privacy since the interviewer and bystanders could overhear.

Some fieldworkers did not offer participants sufficient time or opportunity to explore the app privately due to their time constraints, as they had limited time to complete all households assigned to them within a week. They mentioned that it took longer if participants were left alone to explore the app than if they guided the participants. For example, one fieldworker said: *We should leave a space in between to ensure privacy of our participants*. *If you do so when you come back at a later stage you find that a person is still watching*, *and you have a very limited time and lot of work to do*, *there are many people waiting for you…—*(IDI#03). The time pressure for fieldworkers affected the conduct of the sessions for some participants.

In addition, some participants, particularly older men, required support to navigate the app. Some fieldworkers resorted to selecting stories (characters) for these men based on their perception of what would appeal and be most useful to the user. By doing so, the fieldworker did not give the participants the opportunity to explore the app in private and choose stories for themselves that resonated with them, which may have increased the risk perception and likelihood of a response.

Furthermore, some participants did not receive sufficient exposure to EPIC-HIV 1 because they stopped exploring the app before the end, complaining that it was wasting their time. Some said that they found it was not applicable to their situation. One fieldworker elaborated: *… someone would say I do not see a need for you to play this app…you are wasting my time*. *Rather just test me*, *I do not see a need for this app–*(FGD). In so doing these participants did not access all the information that was included in the app intended to increase their motivation to test.

The food vouchers were easier to deliver compared to EPIC-HIV. The challenges that fieldworkers experienced were related to eligibility criteria for receiving them. The HITS interventions were randomized by communities and yet these communities are in close proximity to each other. This meant that participants in communities that were not assigned to the vouchers would have heard about receiving a voucher when you test for HIV without understanding the eligibility criteria of participating in the trial. Some participants in the communities not randomized to financial incentives expected to receive a voucher once they finished testing and started arguing with fieldworkers on why they were not getting the voucher: *…a person will fight with you and say you’ve tested me*, *but you didn’t give me a voucher*…—(IDI#4 –fieldworker). This was an added burden on the fieldworkers to explain the eligibility for the food vouchers.

Lastly, implementing HITS as part of the HIV surveillance program interacted with the trial and made it difficult to reach some men who did not want to take part in the routine survey. For example, during the FGD, fieldworkers reported that the survey took a long time and participants disliked the sexual behavior questions, which sometimes led to refusals to participate in the HITS trial:

*…we get interrupted because we are doing a lot*, *you find that we are interrupted by the questions about sexual relationship that we ask… They are very long*, *they are annoying to them because they do not like them well…if we can be honest*, *we could say they hate them*. *So*, *you find that even a person is interested*, *he gives- up during the interview because of those questions that were asked*…*—*(FGD).

Thus, men who could have benefited from the trial were missed because of the negative perception of the AHRI HIV surveys.

Also, some participants were in a rush and interested in the HIV test only. In a research context, this is challenging as researchers cannot perform research activities without completing the other research components, including the informed consent which put pressure on them (fieldworkers) to rush procedures:

*…but it’s difficult on males*, *because they are always rushing*. *Sometimes you find that he will come while you haven’t opened the tablet*, *just before you ask anything*, *he will say just do the blood test*. *You will then rush trying to open tablet*, *because you won’t start doing blood test without opening tablet and by that time he will be going and leave you there if you are too slow—*(IDI#4 –fieldworker).

Therefore, where men were only interested in receiving their test result, and not in taking part in the study, the fieldworkers could not complete the study procedures.

The 2^nd^ stage was the linkage to care. Here, the implementation challenges were around the specified time to receive the second voucher and the issue of shared confidentiality within the study team. The eligibility criteria of linking to care within six weeks to receive the second voucher at the clinic was not always clear to participants. For example, participants who linked to care after six weeks did not understand why they were no longer eligible to receive the voucher. One nurse reported that:

*You find a person coming to the clinic to say I was told to request a voucher here*, *only to find that his/her time is overdue…he does not qualify to get that voucher*. *So*, *it was difficult even when you explain to him*, *you could see that he/she does not understand…he thinks you refusing to give him the voucher—*(IDI#2).

The pressure to provide a voucher led to feelings of frustration for the clinic staff.

The study tracker (the experienced fieldworker who delivered EPIC-HIV 2) was sometimes viewed as a person who, through their presence, threatened the participant’s confidentiality. A tracker told us: *He will tell you that he doesn’t want to discuss his status with other people*. Also, he felt pressured to explain his visit to the other members of the household: *Because if you visited a person*, *he would ask why you are visiting him only*, *then you have to explain*. These participants were not comfortable discussing their HIV status with the tracker. There were also participants who were not interested in using the app (EPIC-HIV 2) citing time challenges.

### Mechanisms of action

For the 1^st^ stage -HIV testing- both participants and the implementers found the interventions acceptable and reported that they helped to increase their motivation to test. One participant reflected: *…because one ends up saying let me do the test so that I can get money…*.*—*(IDI#1- financial incentive only). The voucher was viewed to have acted as a catalyst to encourage HIV testing.

Generally, there was a positive reception for the vouchers in the communities. The news of getting a voucher when testing for HIV spread widely within communities and led to participants coming forward and availing themselves for HIV testing. Fieldworkers reported how participants who were initially non-contacts would stop them (fieldworkers) along the road to request that they test them as well. This is what one fieldworker said: *if you didn’t find him/her by the time you arrive at his/her household*, *but you will find him/her on another day waiting for you simply because he/she want to test with an intention to receive voucher*.—(IDI#4). The enthusiasm for the voucher did increase the willingness of some participants to test.

Regarding EPIC-HIV 1, both participants and implementers perceived it to be useful. One participant explained how the app made it easier to understand the importance of knowing his HIV status and helped him make the decision to test: *…they put it in such a way that it clear*, *it was well explained to me and I can say it something that made me end taking a decision to test*.- (IDI#1- both interventions). Participants found the information persuasive and, as in the quote above, described how it motivated them to test.

Fieldworkers also noted how the app was useful in delivering persuasive HIV information to the men. One fieldworker commented in the FGD: `*…you can see that this person would not test but the app has convinced him to test’*. Thus, it was the app that persuaded men to change their minds about testing for HIV.

Both participants and implementers reported that the messages were easily comprehended highlighting how the content and the visuals made it easier to understand/follow the stories. One participant shared how the positive examples of the men in the app resonated with him and helped him to imagine a better future living with HIV: *I can say it encouraged me… the video of a guy who was playing soccer yes*, *he was living with HIV and his girlfriend and every time when he was having sex*, *he used protection*. *He didn’t feel discriminated in community because he has HIV*, *he was living the normal life*.–(IDI#9 –EPIC-HIV only Arm). The men could relate to the people depicted in the app and the messages relayed.

The fieldworkers drew attention to how the stories in the app resonated with the participants and offered participants something more relatable than they could as counselors. Also, implementing EPIC-HIV via an app ensure consistency in delivery: *it makes things easy to see that okay this story of this person is relating to mine because I as a fieldworker or a tracker I would not explain that*. *I would not explain so*, *maybe I would explain about being part of the research study but not touching on that story…- (*FGD). The fieldworkers appreciated the way in which the app helped in relaying messages to participants.

Although some participants complained about the earphones, younger people liked the benefit of receiving earphones as part of the EPIC-HIV intervention. However, this made women, especially young girls that wanted the earphones feel excluded and denied the benefits of participation to receive earphones. This fieldworker told us that: *…the girls they were unable to understand when it comes to a part that doesn’t have headphones*, *when you are about to complete testing process*, *she will ask you as to why I was not given headphones*. *You will then tell her that they were made for the males -(*IDI#3). The focus on men, particularly where the women could see benefits in participation were challenging for fieldworkers to explain.

In the 2^nd^ stage–the linkage to care—some nurses were concerned about using vouchers to motivate participants to link to care. They argued that some participants that came to the clinic were already on HIV treatment and questioning the sustainability of patients coming back to the clinics for treatment refills beyond the intervention. *In linkage it might happen the person was linking because he wants vouchers*, *after linking it might happen he will default because vouchers will be finish—*(IDI#1 nurse). The nurses preferred the app for encouraging participants to continue taking treatment in the long term.

EPIC-HIV 2 participants reflected on how the stories of different characters in the app resonated with the reality of their situation and helped them to make informed decisions about managing their health. A participant who had previously defaulted treatment explained: *The part I have seen as the most essential one for me is where the characters were talking about the risk of defaulting from taking treatment…It motivated me to decide to be re-initiated on treatment…It is really necessary…because characters on the Epic were talking about things they had experienced—(*IDI#2 –EPIC-HIV only Arm). Being able to relate to the characters in the app helped participants to identify with the situations being portrayed–and thereby made the app more acceptable to participants.

### Contextual factors that affected implementation, intervention mechanisms and outcomes

For the 1^st^ stage–HIV testing–although both interventions were perceived to have served as a catalyst to test, the convenience of testing at home further facilitated the acceptance of an HIV test. Participants welcomed the opportunity to be tested at home in their own space which eliminated the barriers associated with going to the clinic to seek HIV testing (time commitments, distance and stigma associated with being seen in the HIV section of a clinic). One participant observed that `*… it is helping us a lot*, *especially for me I am working*, *and you find that I don’t have time to go to the clinic’–*(IDI#4 –financial incentive arm). Furthermore, testing at home provided privacy and opened them up for persuasion to get tested.

In a context where HIV stigma is prevalent, implementing the trial within a household setting could potentially place pressure on household members to conceal their HIV status in order to avoid questions about not receiving the voucher. One fieldworker commented: `*…it’s like others they are asking as to where is yours (participants who did not get a voucher)*, *she doesn’t have anything so*, *you see it becomes a challenge…’ -*(IDI#03). As noted above, giving vouchers only to some people was challenging for the fieldworkers as well as participants.

Participants spoke of the relationship that AHRI has with the community and the HIV work they have been doing in the area, which facilitated the implementation of the AHRI research work, including HITS. However, some people in the community were experiencing research fatigue. For example, one participant commented on how some participants chase away fieldworkers and missed the opportunity to participate in the HITS trial.

For the 2^nd^ stage–linkage to care–some participants highlighted how fear of HIV stigma can act as a barrier to access HIV care despite one being motivated to link to care. They mentioned how some people were afraid of going to the clinic since being seen at the clinic was sometimes associated with living with HIV, particularly with the segregated HIV departments in some clinics. One participant commented: *The fear… I can say a person would be scared to go to the clinic because he fears that people will see him*, *that is what they fear*, *if he goes to the clinic people will laugh at his having this thing as well—*(IDI#5 –financial incentive only). Many men valued privacy, something which could not be guaranteed in the clinic settings.

Additionally, clinic barriers such as long queues and a perception of poor treatment further discouraged people from accessing HIV care. One participant from the financial incentive arm (only) explained how he had not linked to care yet because he always found the clinic full. Delays in linking to care led to a reluctance to link where participants were no longer sure of how to explain the delay. This participant explains how EPIC-HIV 2 had motivated him, but he was no longer sure of what to do: *It gave me motivation*, *I even thought of calling the fieldworker to check what I should do*, *as I had delayed [going to the clinic] for a long time and now the dates in the letter [referral] might clash—*(IDI#3 –EPIC-HIV). The privacy and convenience of being tested at home encouraged some men to test; men who may have been reluctant to go to a clinic or concerned about meeting people who they knew at the clinic.

## Discussion

The process evaluation enabled us to understand why the financial incentives increased uptake of HIV testing but not linkage to care, and why EPIC-HIV did not have an effect on both uptake of HIV testing and linkage to care among men. The extent to which EPIC-HIV, particularly EPIC-HIV 1, was delivered as conceived and planned was poor, explaining the null effect of EPIC-HIV 1 on uptake of HIV testing. EPIC-HIV was developed using different approaches, including a person-based approach and human-computer interaction techniques to ensure that it was usable and engaging for a broad spectrum of end users, however the inconsistent use of earphones, the need for support to navigate the app and time constraints were not anticipated.

Human centered approaches in designing digital health interventions have been described as the most appropriate to ensure fidelity (i.e. that the intervention is delivered as planned) [15, 16]. However, implementation in the real world is complex. The time constraints experienced in our trial mimic what can happen if home-based HIV testing is implemented in a real-world context, highlighting the need to pilot test the interventions, not just the app in isolation, to fully appreciate and plan for the realities of the actual implementation. Other studies have shown the complexity of delivering digital interventions, particularly in resource constrained settings where there is a broad spectrum of end users and low levels of digital literacy with the need to define effective engagement and determine when human support is needed [17].

Financial incentives were easier to implement and acted as catalysts to improve uptake of HIV testing, however the external factors such as fear of stigma and disclosure, as well as perceptions of long queues and poor treatment in clinics acted as barriers and may explain the null effect of both interventions on linkage to care. This underscores the importance of the context and how interventions are mediated by the context to affect outcomes. In our case, the financial incentives and EPIC-HIV could not overcome the external barriers to care despite the internal and external motivation to link to care that the participants reported. This suggests that increasing internal and external motivation may not be ‘sufficient’ and could be more suitable/effective when used to support decentralized HIV care [18, 19]. For example, the convenience of home-based testing facilitated uptake of HIV testing. In addition, the positive examples of men living with HIV portrayed in the app appealed to EPIC-HIV users and helped them to make informed decisions about managing their health. This suggests that offering EPIC-HIV with other interventions such self-testing where end users can explore the app in private in their own time has the potential to encourage men to test and link to care.

This is corroborated by other studies. For example, Hlongwa and colleagues [3] reported inconsistent findings for men to seek confirmatory testing and link to care after self-testing. Travelling costs, long waiting times at the clinics, stigma, discrimination, and privacy concerns were the key barriers to link to care after testing.

### Strengthens and limitations

A limitation is that we interviewed men that received the interventions and therefore we do not know why others did not participate in the trial. Further, the process evaluation was conducted towards the end of the trial and data only analyzed later when we could not make any improvements in the study. Nevertheless, a strength was that the process evaluation helped us to understand how the HITS interventions were implemented, participants responses to them and how the contextual factors interacted with the interventions to affect the trial outcomes.

## Conclusions

Financial incentives increased the uptake of home-based rapid HIV testing and were relatively easy to implement. However, they were not sufficient as a ‘standalone’ intervention to improve uptake of HIV care among men. Furthermore, the barriers such as the fear of stigma and perception of poor treatment and long queues in clinics act prevent men from linking to care. We found, therefore, that because of the implementation and context challenges it was not possible to realize the potential of EPIC-HIV on increasing motivation to test and link to care.
